# T1 mapping using a saturation recovery single-shot acquisition at 3 Tesla MRI in differentiation of normal myocardium from hypertrophic cardiomyopathy

**DOI:** 10.1186/1532-429X-18-S1-P109

**Published:** 2016-01-27

**Authors:** Ryo Ogawa, Tomoyuki Kido, Masashi Nakamura, Teruhito Kido, Akiyoshi Ogimoto, Masao Miyagawa, Teruhito Mochizuki

**Affiliations:** 1 Ehime University Graduate School of Medicine, Toon city, Japan; 2 Saiseikai Matsuyama Hospital, Matsuyama, Japan

## Background

Background Diffuse myocardial fibrosis is characteristic feature of hypertrophic cardiomyopathy (HCM). T1 mapping may enable non-invasive evaluation of diffuse myocardial fibrosis in HCM. There have been many studies that T1 mapping using a modified Look-Locker inversion recovery (MOLLI) sequence. However, there have been few studies using a SASHA for T1 mapping at 3 Tesla MRI. The purpose of this study was to examine T1 values using a SASHA, in the differentiation between healthy controls and HCM patients.

## Methods

The clinical diagnosis of HCM was established via echocardiography, Cardiac Magnetic Resonance, electrocardiogram, laboratory examination, family history and other clinical data. Twenty patients with HCM and ten healthy controls, underwent left-ventricular T1 mapping in 3 short-axis slices (basal, mid, apex) at 3 Tesla MRI (Philips Achieva). For T1 mapping, SASHA was used before and at 10 minutes after injection of 0.1 mmol/kg of gadolinium contrast. T1 values were quantified for 4 segments (anterior, lateral, septum, inferior) of 3 short-axis slices.

## Results

Native T1 values were significantly longer in HCM compared with healthy controls (HCM 1377 ± 72 ms vs healthy controls 1285 ± 49 ms; p < 0.01). Post-contrast T1 values were significantly shorter in HCM compared with healthy controls (HCM 840 ± 64 ms vs healthy controls 908 ± 94 ms; p < 0.01). A cutoff value of 1363 ms for native T1 values allowed differentiation between healthy and abnormal segments (HCM) with a sensitivity of 57%, specificity of 92%, accuracy of 69%, and an area under the curve (AUC) of 0.77. On the other hand, post-contrast T1 values showed lower discriminative ability than native T1 values.

## Conclusions

Native T1 values using a SASHA provide better distinction between healthy controls and HCM. Native T1 values using a SASHA have a potential to detect myocardial fibrosis in HCM without using gadolinium contrast agents.

**Table 1:** T1 values in HCM and healthy controls

	native T1 values (ms) (median ± quartile deviation)	post T1 values (ms) (median ± quartile deviation)
HCM (240 segments)	1377 ± 72	840 ± 64
healthy controls (120 segments)	1285 ± 49	908 ± 94

**Figure 1:**
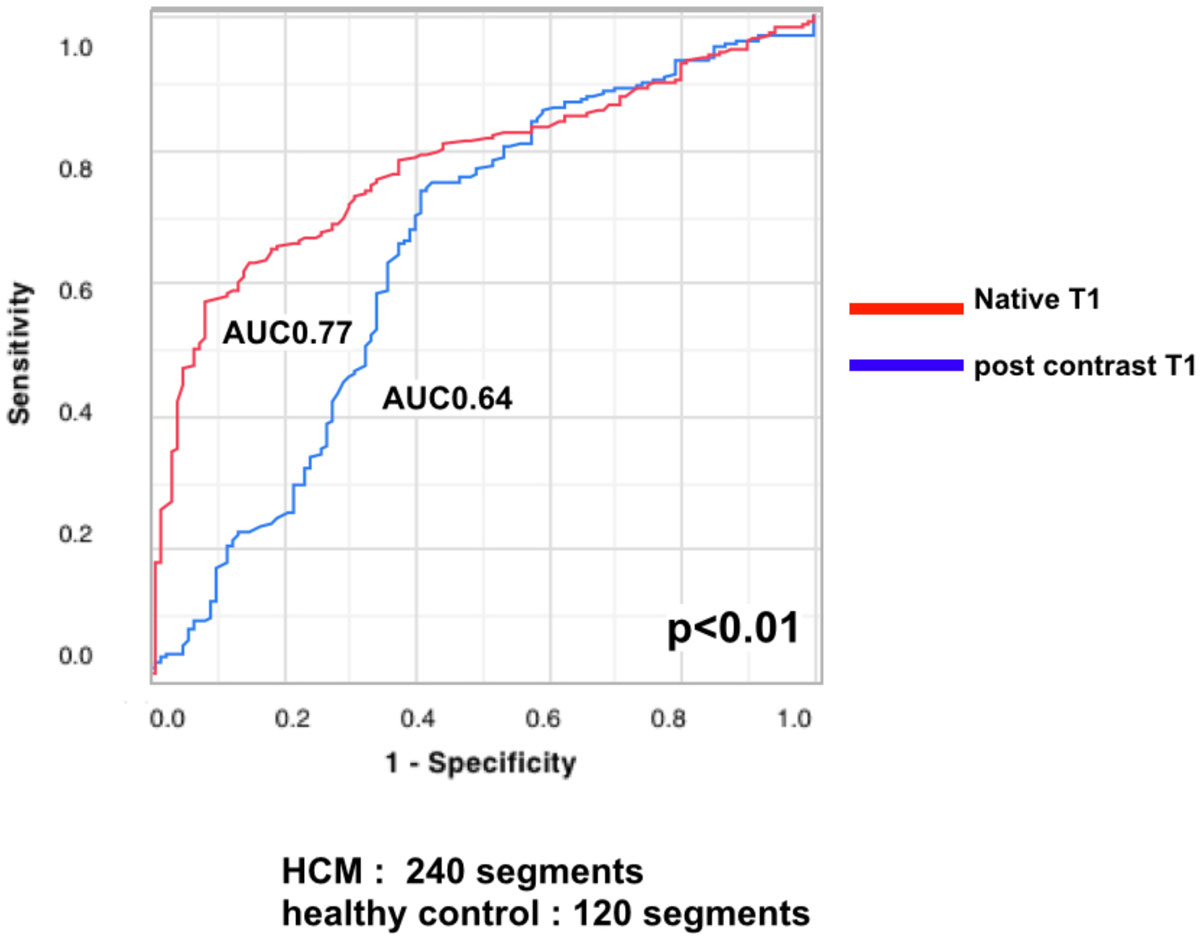
**ROC curves for T1 values in differentiation between healthy and abnormal segments (HCM)**. Native T1 values using a SASHA provide better distinction between healthy and abnormal segments (HCM) than post-contrast T1 values.

